# Cotargeting TREM2 and IL2 pathways triggers multipronged anticancer immunity

**DOI:** 10.1002/1878-0261.70210

**Published:** 2026-01-21

**Authors:** Isaure Vanmeerbeek, Jenny Sprooten, Abhishek D. Garg

**Affiliations:** ^1^ Laboratory of Cell Stress & Immunity (CSI), Department of Cellular & Molecular Medicine, KU Leuven Belgium

**Keywords:** cancer immunotherapy, immunocytokines, myeloid checkpoints, tumour‐associated macrophages

## Abstract

Triggering receptor expressed on myeloid cells 2 (TREM2) has emerged as a key immunosuppressive target on tumour‐associated macrophages (TAMs), where it coordinates protumorigenic and anti‐inflammatory functions within the tumour microenvironment (TME). Unfortunately, recent clinical evidence indicates that therapeutic TREM2 blockade has suboptimal efficacy in cancer patients. Now, Von Locquenghien et al. report that MiTE‐144, a TREM2 blocking antibody fused to an IL2 variant with TME‐restricted activation, demonstrates superior anticancer efficiency compared to TREM2 blockade alone in the preclinical setting. Importantly, MiTE‐144 showed reduced systemic inflammation or hepatotoxicity relative to TREM2 blockade and/or ‘generic’ IL2 immunocytokine approaches. Detailed TME analysis of MiTE‐144‐treated tumours showed substantial reprogramming of the myeloid compartments, together with activation of NK/CD8^+^ T cells. While this study tackled several limitations of anti‐TREM2 monotherapy, more attention is needed towards clinically relevant immunotherapy barriers in therapy‐refractory tumour settings.

## Graphical abstract

Von Locquenghien *et al*. report that MiTE‐144, a triggering receptor expressed on myeloid cells 2 (TREM2) blocking antibody fused to interleukin‐2 (IL2) variant with tumour microenvironment restricted activation, demonstrates superior anticancer efficiency in a preclinical setting. MiTE‐144‐treated tumours showed reprogramming of the myeloid compartments, together with activation of NK/CD8+ T cells.

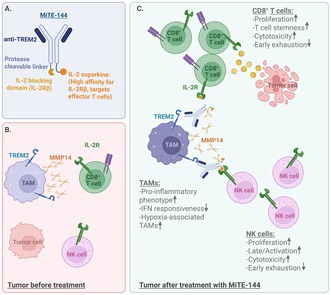

**Abbreviations**
ALT: alanine transaminase
AST: aspartate transaminase/aminotransferase
CAR‐T cell: chimeric antigen receptor T cell
CSF1R: Colony‐Stimulating Factor 1 Receptor
CTLA4: Cytotoxic T‐Lymphocyte‐Associated protein 4
ICI: immune‐checkpoint inhibitors
ICK: immunocytokine
IL: interleukin
IL‐2Rβ: interleukin 2 receptor subunit beta
MiTE: myeloid‐targeted immunocytokines and natural killer/T cell enhancer
MMP14: macrophage‐specific matrix metalloprotease 14
NK cell: natural killer cell
PD1: programmed cell death protein 1
PDL1: programmed death ligand 1
PDTF: patient derived tumour fragment
RCC: renal cell carcinoma
SIRPα: signal regulatory protein alpha
TAM: tumour‐associated macrophages
TCR: T cell receptor
TIL: tumour‐infiltrating lymphocyte
TIM3: T‐cell immunoglobulin and mucin domain‐containing protein 3
TME: tumour‐microenvironment
TREM2: triggering receptor expressed on myeloid cells 2
VISTA: V‐domain immunoglobulin suppressor of T cell activation

Cancer immunotherapy has improved the prognosis of many patients for specific cancer types, but several other cancer types remain resistant [1, 2]. A plethora of immunotherapies exist today that primarily focus on activating T‐cell‐mediated anticancer immunity, for example immune‐checkpoint inhibitors (ICI), chimeric antigen receptor (CAR)‐T cells [3], T‐cell receptor (TCR)‐based therapies [4], bispecific antibodies [5] and tumour‐infiltrating lymphocyte (TIL) therapies [6]. Some of these have had better success against haematological malignancies than solid tumours, primarily owing to the latter's heterogenous antigenicity, clonal or subclonal heterogeneity and/or immunosuppressive tumour microenvironment (TME) [7]. Moreover, besides activation of the T cells via some of these immunotherapies, sustaining their expansion, differentiation and survival is also essential to create long‐lasting immune responses. Cytokines such as IL2, IL7 and IL12 play an essential role in T‐cell biology and immune cell function, which has encouraged combinatorial strategies integrating cytokine‐based interventions [7, 8]. Recent development of antibody‐cytokine fusion proteins or immunocytokines (ICKs) has shown the potential to enhance T‐cell‐mediated tumour immunity without off‐target toxicity and widespread pleotropic effects of cytokines [9]. Currently, these are being combined or synthetically linked with T‐cell targeting ICIs such as anti‐PD(L)1 and anti‐CTLA4 [10, 11]. Indeed, ICKs such as anti‐PD1‐IL2 or PD1‐IL15 are in preclinical or phase 1 clinical trials [7], with promising results [12, 13]. However, some issues remain to be resolved including (but not limited to) side effects/toxicities [13], overcoming the immunosuppressive TME dominated by tumour‐associated macrophages (TAMs) [14, 15] and T‐cell‐mediated escape mechanisms [16, 17].

TAMs indeed represent an insurmountable immunotherapy barrier. Multiple therapies have undergone testing to inhibit immunosuppressive TAMs by targeting either immune‐checkpoints on TAMs (such as PD‐L1, TIM3, VISTA) or immunosuppressive TAM pathways (such as CSF1R, CD163, IL10, IL4, SIRPα) with mixed success or outright failures [14, 15, 18, 19, 20]. This has called for discovery and targeting of additional TAM‐specific targets. Herein, triggering receptor expressed on myeloid cells 2 (TREM2) has recently emerged as a major immunosuppressive target on TAMs coordinating their protumorigenic or anti‐inflammatory activity in the TME. TREM2 expression is predominantly tumour‐specific and correlates with poor response to ICI [18, 21, 22]. However, preliminary results of a first‐in‐human phase 1a/1b study of the anti‐TREM2 antibody, PY314, showed modest antitumour activity against advanced solid tumours [22]. Even combinatorial treatment with anti‐PD1 did not meet the efficacy threshold, leading to early termination of this clinical trial. These disappointing results echo failures of several such TAM targeting therapies and call for ‘smarter’ immunotherapy modalities [23].

To address these issues, Von Locquenghien et al. focussed on overcoming the limited therapeutic effect of TREM2 targeting antibodies. Spatial and single‐cell analysis of human breast, lung and colon samples highlighted the importance of the interaction between TAM and T cell in human cancers. Guided by these insights, Von Locquenghien et al. sought to simultaneously activate the TAM compartment as well as effector T cells by designing a TREM2 blocking antibody fused to an IL2 variant with high affinity for IL2Rβ. Use of this engineered IL2 variant preferentially promoted effector T‐cell activation over regulatory T‐cell (Treg) activation. Although both TREM2 blockade and IL2 pathway activation were functionally validated *in vitro*, the *in vivo* use of their myeloid‐targeted immunocytokines and natural killer (NK)/T cell enhancers (MiTEs) proved suboptimal. Mouse studies revealed significant adverse effects, most likely due to the systemic activity of IL2.

Motivated by these challenges, the investigators developed an alternative format designed to block the IL2–IL2Rβ interaction outside the TME (MiTE‐144). This approach incorporated a blocking domain linked via a peptide that was cleavable by the tumour macrophage‐specific matrix metalloprotease 14 (MMP14), thereby restricting IL2 activation to the tumour while preserving MiTE potency. MMP14 expression was confirmed to be higher in tumour tissue rather than in normal adjacent tissue in both human and mouse samples. Importantly, *in vivo* administration did not induce systemic inflammation or hepatotoxicity, as evidenced by stable body weight, unchanged systemic cytokine levels, normal ALT/AST enzyme levels and absence of spleen enlargement. Additionally, biodistribution analysis showed preferential enrichment of the MiTE‐144 antibody in the tumour rather than in nontarget tissues.

The MiTE‐144 treatment alone resulted in almost a complete response in the highly immunogenic MC38 tumours, while combination therapy with anti‐CTLA4 eradicated tumours in 6 out of 7 mice. Differential gene expression analysis on the treated tumours indicated that this high efficacy was driven by the reprogramming of the TAM and dendritic cell (DC) compartments, together with NK and CD8^+^ T cells. Curiously, MiTE‐144 treatments induced a strong perturbation of the myeloid compartment, characterised by a reduction in IFN‐responsive and an expansion of hypoxia‐associated TAMs. This atypical modification of the myeloid compartment probably requires more future research. Within the CD8^+^ T cells and NK cells, a broad molecular reprogramming was observed, including upregulation of proliferation, activation, cytotoxic and proliferation genes accompanied by downregulation of early exhaustion genes. Despite the MiTEs' IL2 variant favouring the CD8^+^ T cells, a slight Treg activation was observed. These *in vivo* findings were further supported by analysis of patient derived tumour fragments (PDTFs) treated with MiTE‐144, which showed an increase in CD8^+^ T cell and NK cell activation and proliferation.

In summary, the authors developed MiTEs, myeloid‐targeted ICKs, designed to achieve dual immune activation. The integration of TREM2 antagonism with protease‐restricted IL2 in *trans* reprogrammed TAMs and triggered NK/T cell effector functions. Surprisingly, the authors reported an increase in hypoxia‐associated and a decrease in IFN‐responsive TAMs in tumours that responded to therapy. This is somewhat paradoxical, as hypoxia has been linked to wound‐healing macrophage phenotypes and reduced antigen presentation [24]. Consequently, hypoxia‐associated TAMs are generally associated with poor cancer prognosis [25]. In contrast, inflammatory or IFN‐responsive TAMs have previously been linked to a better prognosis [26]. This highlights the context‐dependant nature of TAM phenotypes.

The approach of prioritizing a tumour‐restricted cytokine activation tackled several limitations of prior ICKs, including toxicity issues, limited durability due to TME‐driven immune dysfunction and T‐cell‐mediated escape mechanisms albeit in relatively T‐cell ‘supportive’ tumour models. These findings are fundamentally promising and warrant further translational and preclinical testing from clinical efficacy perspective, for example, long‐term safety of the protease‐activated IL2 should be tested in nonhuman primates. Notably, their use of a cleavable domain to minimise off‐target ICK activity may be applicable to a broader range of next‐generation ICKs. The activity and antitumour efficacy of MiTE‐144 across therapy‐refractory tumour types should be explored, as well as its combination with conventional anticancer therapy and other immunotherapies. Rational selection of cytokine cargo might also further enhance efficacy [8, 26].

## Conflict of interest

The authors declare no conflict of interest.

## Author contributions

IV and JS wrote the commentary. ADG provided senior supervision, critically re‐wrote/edited the commentary and refined the language.

## Data Availability

Data sharing is not applicable to this article as no new data were created or analysed in this study.
